# Dissecting the roles and clinical potential of YY1 in the tumor microenvironment

**DOI:** 10.3389/fonc.2023.1122110

**Published:** 2023-04-04

**Authors:** MengNa Li, JianXia Wei, ChangNing Xue, XiangTing Zhou, ShiPeng Chen, LeMei Zheng, YuMei Duan, HongYu Deng, Wei Xiong, FaQing Tang, GuiYuan Li, Ming Zhou

**Affiliations:** ^1^ Key Laboratory of Carcinogenesis, National Health Commission, Hunan Cancer Hospital and the Affiliated Cancer Hospital of Xiangya School of Medicine, Central South University, Changsha, China; ^2^ Cancer Research Institute, Central South University, Changsha, China; ^3^ The Key Laboratory of Carcinogenesis and Cancer Invasion of the Chinese Ministry of Education, Central South University, Changsha, China; ^4^ Hunan Key Laboratory of Oncotarget Gene, Hunan Cancer Hospital and the Affiliated Cancer Hospital of Xiangya School of Medicine, Central South University, Changsha, China; ^5^ The First Clinical College of Changsha Medical University, Changsha, China

**Keywords:** YY1, cancer, tumor microenvironment, tumor progression, tumor immune

## Abstract

Yin-Yang 1 (YY1) is a member of the GLI-Kruppel family of zinc finger proteins and plays a vital dual biological role in cancer as an oncogene or a tumor suppressor during tumorigenesis and tumor progression. The tumor microenvironment (TME) is identified as the “soil” of tumor that has a critical role in both tumor growth and metastasis. Many studies have found that YY1 is closely related to the remodeling and regulation of the TME. Herein, we reviewed the expression pattern of YY1 in tumors and summarized the function and mechanism of YY1 in regulating tumor angiogenesis, immune and metabolism. In addition, we discussed the potential value of YY1 in tumor diagnosis and treatment and provided a novel molecular strategy for the clinical diagnosis and treatment of tumors.

## 1 Introduction

Within the tumor microenvironment (TME), cancer cells coexist with noncancerous adjacent cells and components that constitute the TME and impact tumor growth and progression through diverse mechanisms. In addition to cancer cells, the TME comprises immune cells, fibroblasts, bone marrow-derived inflammatory cells, as well as noncellular components such as abnormal tumor blood vessels, signaling molecules, and extracellular matrix (1, 2). As the forces of the tumor cells grow, the microenvironment that originally suppressed tumor growth and protected the survival of normal cells has also been gradually transformed into a place suitable for tumor cells to survive. Furthermore, the microenvironment provides essential nutrients, amino acids, and nucleotides to the tumor, facilitating tumor growth. At the same time, it forms a protective barrier against the pernicious effect of immune cells and drugs during tumor treatment (3). Yin-Yang 1 (YY1) is a ubiquitous transcription factor with multiple roles in tumorigenesis, functioning as both a transcriptional activator and repressor (4, 5). Moreover, its function depends on its interacting partners, promoter environment, and chromatin structure (6, 7), thereby regulating transcriptional activation and repression of approximately 10% of the total human gene set associated with a variety of cellular biological processes (8, 9). Several recent reports suggest that YY1 mostly acts as an oncogene in most types of cancers but also acts as a tumor suppressor in some other types of cancer (10–13). Therefore, this review focuses on the expression patterns of YY1 in different tumors, its influence on tumor angiogenesis, tumor metabolism and the tumor immune microenvironment, and its potential value in tumor diagnosis and treatment. This review expands the understanding of the roles and mechanisms of YY1 in the TME and provides a novel molecular strategy for the clinical diagnosis and treatment of tumors.

## 2 Expression of YY1 in different tumors

YY1 is widely expressed in various tissues, and its upregulation in different types of tumors shows different clinical implications. To investigate the differential expression of YY1 in different types of tumors, studies have analyzed GEO datasets and found that YY1 has high mRNA expression in most tumor tissues (14). Likewise, several studies have shown that YY1 is highly expressed in breast cancer (15), bladder cancer (16), colorectal cancer (17), cervix cancer (18), esophageal carcinoma (19), gastric cancer (20), glioma (21), hodgkin lymphoma (22), hepatocellular carcinoma (23), kidney cancer (24), lung cancer (25), melanoma (26), osteosarcoma (27), ovary cancer (28), and prostate cancer (29). In addition, in the studies evaluated for all paired primary and metastatic samples, YY1 expression appeared to be increased in metastatic tissues compared with matched primary tumor tissues. These results indicate that YY1 is closely related to tumor invasion and metastasis and is most likely involved in regulating the TME. More intriguingly, In osteosarcoma. YY1 presents lower expression levels in pediatric osteosarcomas compared to normal human osteoblasts (30). In addition, YY1 is also not always highly expressed in tumors, such as melanoma, YY1 expression is significantly lower in metastatic melanoma than in normal melanocytes (31). Furthermore, we have also recently revealed an additional contribution of YY1 in nasopharyngeal carcinoma (NPC). Compared with the expression of YY1 in normal NPE tissues and NPC tissues, most patients exhibited a significant decrease, and its expression negatively correlated with increasing clinical TNM stage and positively correlated with the OS rate with NPC (13). It is same with pancreatic ductal adenocarcinoma (PDAC), several reports indicate that YY1 is downregulated in PDAC tissues compared with adjacent normal pancreatic tissues, and the high expression of YY1 represents a better prognosis in patients with PDAC (32). These results suggest that YY1 has two distinct expression patterns (high or low) in different tumor types, and playing a dual role as an oncogene or tumor suppressor to facilitate tumorigenesis or tumor progression (**Table 1**).

**Table 1:** Expression profile and function of YY1 in a variety of tumors.

Expression	Cancer type	Function	Ref.
up regulation	breast cancer	inhibits	(33, 34)
		promotes	(15)
	bladder cancer	promotes	(16)
	colorectal cancer	promotes	(17)
	cervix cancer	promotes	(18)
	esophageal carcinoma	inhibits	(19)
	gastric cancer	promotes	(20)
	glioma	promotes	(21)
	hodgkin lymphoma	promotes	(22)
	hepatocellular carcinoma	promotes	(23)
	kidney cancer	promotes	(24)
	lung cancer	promotes	(25)
	melanoma	promotes	(26)
	osteosarcoma	promotes	(35)
	ovary cancer	promotes	(28)
	prostate cancer	promotes	(29)
down regulation	PDAC	inhibits	(32, 36)
	NPC	inhibits	(13)

## 3 Role of YY1 in the tumor microenvironment

### 3.1 YY1 in tumor angiogenesis

Tumor vasculature is a critical component of the TME, which is the critical channel of oxygen and nutrient delivery for maintaining tumor growth but is also a pathway of tumor cell metastasis (37). Due to insufficiencies in the tumor vasculature, tumor cells are often exposed to a hostile TME with low nutrient and oxygen levels (38). Tumor cells secrete angiogenic factors to induce neovascularization to satisfy tumor cell oxygen and nutrient requirements (39). Vascular endothelial growth factor A (VEGFA) is the most critical and primary component of the VEGF family, which is a multipurpose cytokine active on blood vessel cells (40). Through a paracrine mechanism induces tumor vascularization to meet the increased requirement of oxygen and nutrients and remove metabolic wastes (41, 42). VEGF is usually regulated by HIF-1α or the CXCR4/SDF-1 axis in tumors (43). The molecular mechanism between the YY1 and VEGF was discovered in multiple studies. De et al. found that YY1 was a critical component of the HIF-1α complex and binds its target sequences on the regulatory regions of VEGFA, -B, and -C to upregulates the transcriptional activity and expression of them (44). In addition, studies have shown that the expression of YY1 was upregulated in the malignancy of osteosarcoma, and silencing the expression of YY1 can significantly downregulate the activation of the VEGF/CXCR4 axis, thereby inhibiting the angiogenesis, invasion and metastasis (45). HCC is a well-known typical angiogenesis-dependent solid tumor with rich blood vessels, and YY1 is reported to be involved in regulating tumor malignancy in HCC. Yang et al. found that microvessel density (MVD) was positively correlated with YY1 and poor prognosis in HCC, and overexpression of YY1 promoted VEGFA transcriptional activity by binding to the VEGFA promoter (46). These results highlight a new mechanism by which YY1 plays an essential role though inducing VEGFA transcription in HCC angiogenesis. In addition to direct regulation, the indirect regulation between YY1 and VEGF has gradually been discovered. Li et al. found that YY1 contributes to angiogenesis *via* SNHG5/miR-26b/CTGF/VEGFA axis in AML (47). Moreover, a VEGF-independent mechanism to promote angiogenesis was discovered. In the presence of KRAS mutations, YY1 promoted neovascularization by targeting the ZNF322A promoter to increase its expression. It is significant to note that ZNF322A increases the sonic hedgehog (Shh) expression, which encodes a secreted factor that turns on pro-angiogenic responses in endothelial cells. According to these results, disruption of the Kras/YY1/ZNF322A/Shh transcriptional axis encourages lung cancer neoangiogenesis and cancer development (48). Not only protein-encoding genes but YY1 can target the transcription of lncRNA MCM3AP-AS1, further targeting the miR-340-5p/KPNA4 axis stimulated lung cancer angiogenesis and progression (49). Similarly, in cholangiocarcinoma, it is also found that the Circ-CCAC1/miR-514a-5p/YY1/CAMLG axis disrupts endothelial barrier integrity and induces angiogenesis (50).

YY1 plays dual biological roles in the initiation and progression of various cancers. Likewise, YY1 exhibits promote or inhibit angiogenesis. Several previous studies have reported that YY1 plays a tumor suppressor role in PCDA. For example, YY1 inhibited angiogenesis by downregulating the TPPP-mediated p38/MAPK and PI3K/AKT pathways opening a new horizon in PCDA research. ChIP sequencing results showed that YY1 directly binds to the promoter region of TPPP and thus inhibits pancreatic cancer cell migration, invasion and angiogenesis (51). Beyond that, latterly the discovery of YY1 suppressed the invasion and metastasis of PCDA cells by downregulating the expression of MMP10 through the MUC4/ErbB2/p38/MEF2C regulatory axis (12, 32). Overall, this is strong evidence that YY1 plays an inhibitory role in neo-angiogenesis.

**Figure 1:**
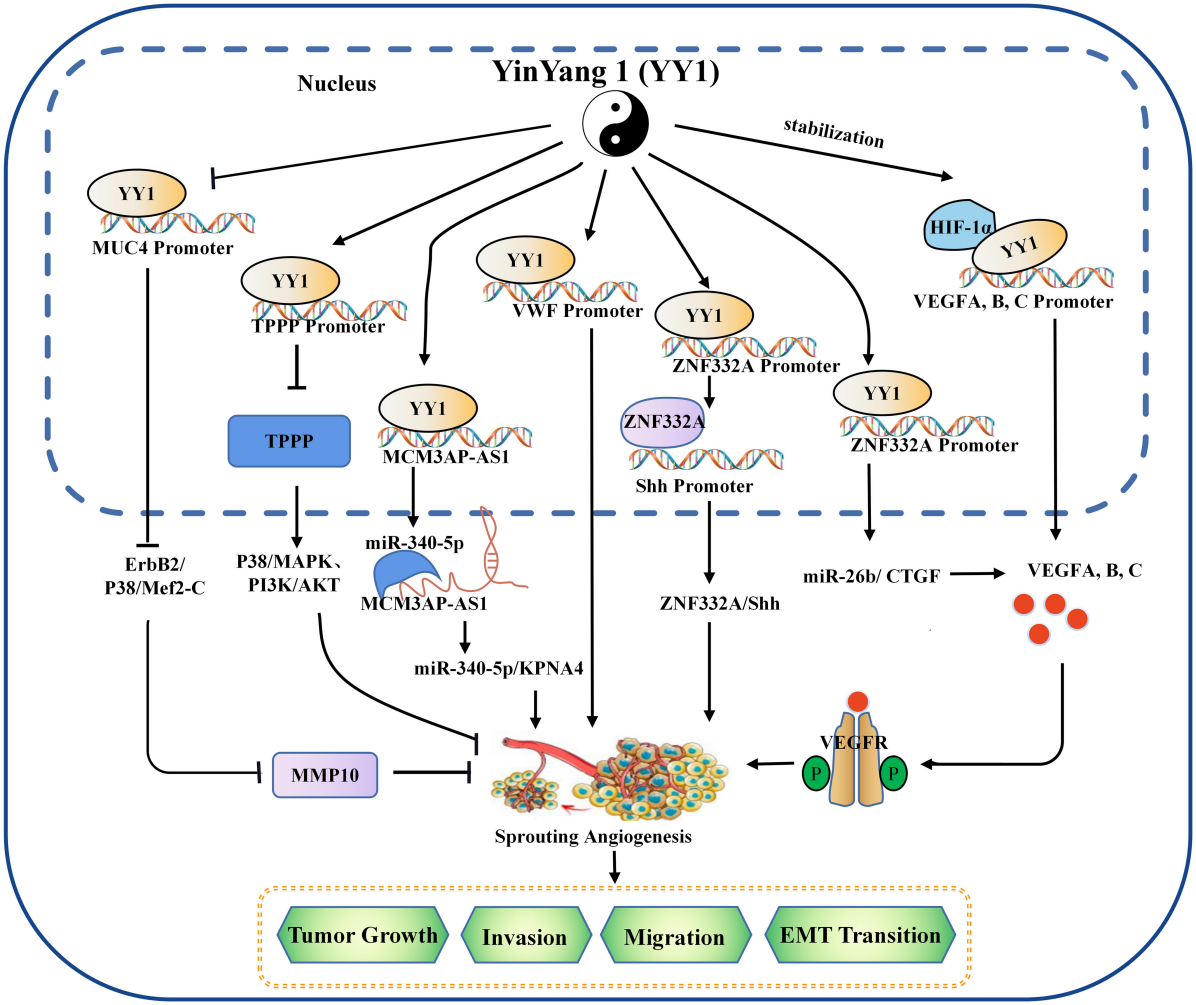
The mechanism of YY1 regulating angiogenesis in the tumor microenvironment. YY1 regulates the expression of crucial proteins associated with tumor neovascularization (such as SNHG5, VWF, MUC4, ZNF332a, TPPP, MCM3AP-AS1, VEGF family, et al.), by interacting with their promoters, thus to regulate the proliferation, migration, invasion, and EMT transition of cancer cells in an oncogene or tumor suppressor role. YY1, Yin Yang 1; HIF-1α, hypoxia-inducible factor 1-α; VEGF, vascular endothelial growth factor; VEGFA, vascular endothelial growth factor A; TPPP, tubulin polymerization promoting protein; MMP 10, matrix metallopeptidase 10; EMT, epithelial-mesenchymal transition.

In general, existing evidence indicates that YY1 acts as a double-edged sword that alters the TME by promoting or inhibiting angiogenesis in different tumor types (**Figure 1**), where some of the position of YY1 regulating target genes related to angiogenesis occur in cancer cells, which indirectly affected the formation of blood vessels by regulating the expression of angiogenesis related factors. Some regulation positions take place in vascular endothelial cells and directly affects the formation of blood vessels. The effect and mechanism of YY1 on angiogenesis in various tumors are shown in **Table 2**.

**Table 2:** The target gene and mechanism of YY1 on angiogenesis.

Cancer type	Target gene/regutation	Position of regulation	Function	Ref
AML	SNHG5/up	cancer cells	promotes	(47)
melanoma	VEGF/up	vascular endothelial cells	promotes	(52)
osteosarcoma	VEGF/up	cancer cells	promotes	(53)
HCC	VEGFA/up	vascular endothelial cells and cancer cells	promotes	(46)
lung cancer	ZNF322A/up	cancer cells	promotes	(48)
lung cancer	MCM3AP-AS1/up	cancer cells	promotes	(49)
PCDA	TPPP/down	cancer cells	Inhibits	(51)
PCDA	MUC4/down	cancer cells	Inhibits	(12)
cholangiocarcinoma (CCA)	CAMLG/up	vascular endothelial cells and cancer cells	promotes	(50)
CRC	NA	vascular endothelial cells and cancer cells	promotes	(54)
breast cancer	VWF/up	cancer cells	promotes	(55)

### 3.2 YY1 in immunity

Human cells are constantly under surveillance by the immune system. The TME is the main battleground between tumor cells and the host immune system, and tumors can escape immune surveillance by initiating various immunosuppressive cells or establishing an immunosuppressive microenvironment (56, 57). Recent studies have shown that YY1 regulates broad general processes throughout all stages of T-cell and B-cell differentiation (58, 59).

#### 3.2.1 T cells

Immune checkpoint activation is a critical mechanism by which tumor cells evade clearance by immune cells. In the TME, where tumor cells are located, the surface antigens of tumor cells cannot be presented to T cells, resulting in tumor cells evading immune killing. Programmed death receptor-1 (PD-1) and cytotoxic T lymphocyte-associated antigen-4 (CTLA-4) are the most common immune checkpoint receptors expressed on the surface of T lymphocytes (60). YY1 has been shown to positively regulate immune checkpoint receptors such as PD1 and CTLA-4 (61), and YY1 was upregulated in PD1-positive T cells infiltrating lymphocytes in melanoma tumors and directly regulated the expression of PD1 and LAG3 by binding to the promoter region (62, 63). PD-1 is a critical receptor expressed on activated T cells that inhibits T cell-mediated immune responses upon binding to its ligand programmed cell death ligand 1 (PD-L1) (64). YY1 can increase PD-L1 expression through various signaling pathways. As a negative regulator of p53, YY1 inhibits the expression of miR-34a downstream of p53, while downregulation of miR-34a expression leads to an increase of PD-L1 expression by binding to the PD-L1 3’UTR (65). YY1 has also been shown to promote PD-L1 expression by downregulating PTEN *via* p53 and activating the PI3K/Akt/mTOR pathway (66). YY1 has been reported to be associated with T cell apoptosis. FGL1 promotes the secretion of interleukin-2 by T cells and induces their apoptosis. Indeed, YY1 is the upstream molecule of FGL1, which was found to be transcriptionally regulated by YY1 (67). These results suggest that YY1 may hinder T-cell-mediated tumor immunotherapy by interfering PD-1/PD-L1 checkpoint.

**Figure 2:**
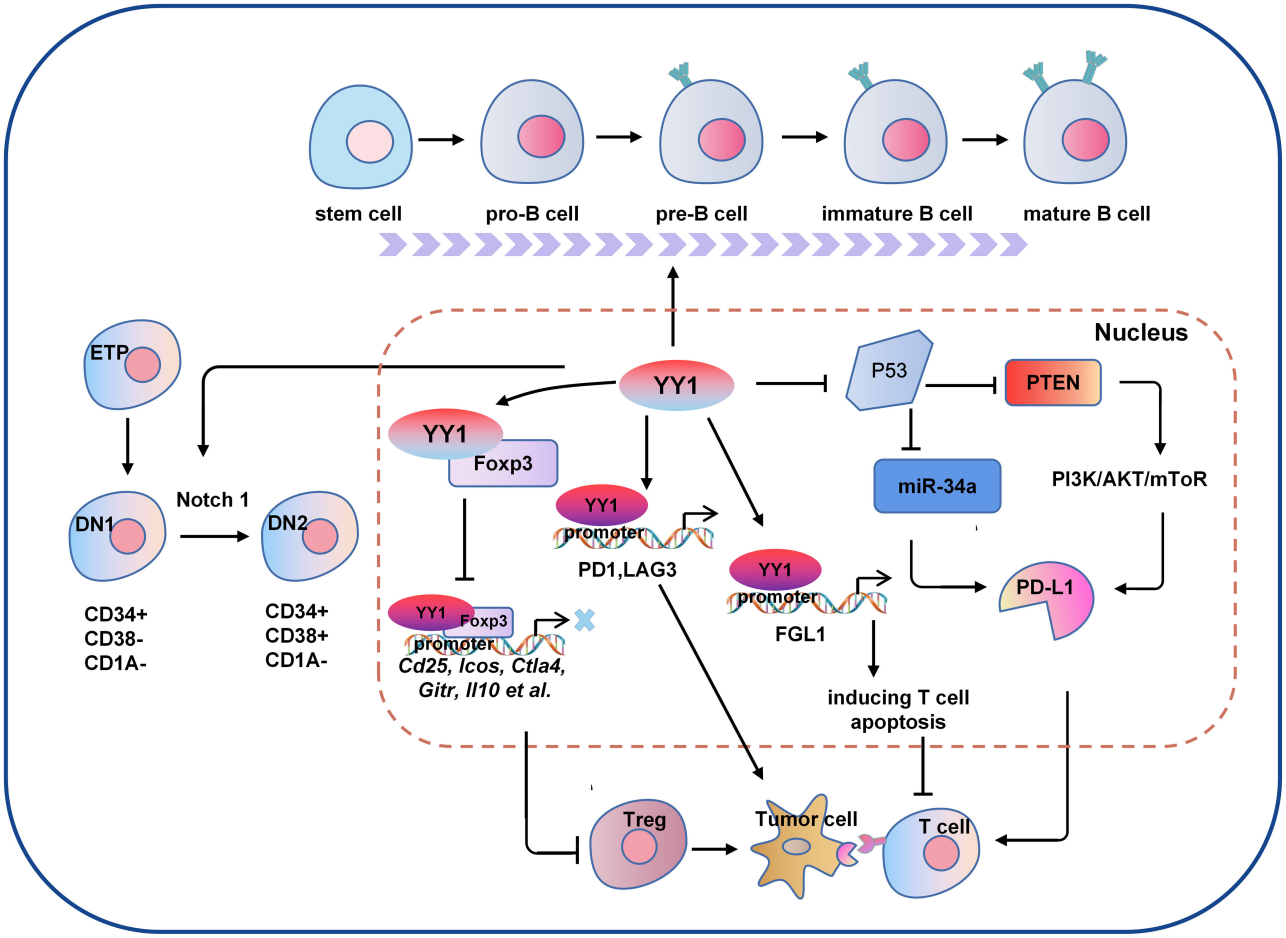
The molecular mechanism of YY1 regulating B cell and T cell-mediated tumor immunity. Several signaling pathways crosstalk exist between the regulations of YY1 and T cell function. For example, YY1 inhibits the function of T cells by blocking Foxp3 mediated transcription of its downstream genes Cd25, Icos, Ctla4, Gitr and Il10 et al., or directly regulating the expression of PD1 and LAG3 by binding to their promoter regions. In addition, YY1 promotes the DN1-to-DN2 T cell transition by activating Notch1 pathway. For B cells, YY1 is essential for all stages of B cell differentiation, thus to be involved in B cells-mediated immune escape. DN1, double negative 1; DN2, double negative 2; PD-L1: programmed death ligand 1; Foxp3, forkhead box p3.

As the tumor progresses, the TIME is constantly changing, and the speed of tumor development depends on the proportion and characteristics of T cells within the TIME. As early as ten years ago, evidence in the field of tumor immunity suggested that the TME limits the accumulation of T cells among tumor cells. Regulatory T (Treg) cells are essential for maintaining immune homeostasis (60, 68). Hwang et al. showed that the expression of YY1 in Treg cells was lower than that in Tconv cells, and the overexpression of YY1 led to a significant decrease in the expression of Foxp3, making the suppressive function of Treg cells ineffective (69). As one of the most concerned phenotypes in the CD4 T cell family, Treg cells are an immunosuppressive subtype in nature, but with a dual personality as an angel side, Treg cells can regulate the immune system, inhibit the pathological immune response caused by the overactivation of autoreactive T cells, and control inflammation. It plays a key role in maintaining autoimmune tolerance and immune homeostasis. However, in the tumor microenvironment, it shows a devil side. It inhibits the proliferation, metabolism and killing function of CD8 T cells by secreting inhibitory factors such as IL-10, TGF-β and affecting the maturation of dendritic cells (DC). It induces the immune escape of tumor cells by inhibiting the anti-tumor immune response, thus promoting the growth and proliferation of tumors (70). Foxp3 is the critical transcription factor for Treg cell function and differentiation (71). YY1 physically interacts with Foxp3 to directly switch on Foxp3 target genes and interfere with Foxp3-dependent target gene expression. Thus, YY1 inhibits the Treg cell’s differentiation and function by blocking Foxp3 (69, 72). YY1 is a polycomb group (PcG) protein and an original member of the local group of sequence-specific DNA-binding PcG proteins in mammals (73). PcG proteins silence target genes through histone modification, especially methylation modification (74). By contrast, YY1 promotes early T cell survival through its PcG function. In the thymus, T cell development begins when double negative (DN) progenitor T cells lacking CD4 and CD8 expression become CD4^+^CD8^+^ double positive pre-T cells (DP) and then differentiate into mature single CD4^+^ or CD8^+^ positive cells, DN cells have four stages (75–77). Ana et al. demonstrated that YY1 promotes DN1 to DN2 T cell transition and early T cell survival independent of PcG/REPO domain function (78). Overall, YY1 is crucial for T-cell differentiation, function, and development, but YY1 has opposite effects on T-cell development and function (**Figure 2**).

#### 3.2.2 B cells

B lymphocytes are derived from bone marrow hematopoietic stem cells through a series of differentiation stages. Activated B cells produce many cytokines involved in immune regulation, the inflammatory response and hematopoiesis (79). YY1 plays an important role in Ig rearrangement and B-cell development. Studies have shown that conditional knockdown of YY1 prevented the transition of pro-B cells to cells in the pre-B-cells and early developmental stages of B cells (58). Subsequent proof found that YY1 regulates the germinal center B-cells (GC B cells) transcriptional program (59). In specific deletion of YY1 in GC B cells, B-cell numbers in both unimmunized and immunized conditions are severely reduced. In addition, YY1 also affects the immunoglobulin class switch recombination (CSR) process in splenic B cells. These studies strongly suggest that YY1 is essential for B cell differentiation in all stages and is required for the survival and proliferation of B cells (**Figure 2**), B-NHL is a heterogeneous lymphoma derived from a B-cell mutation (80). Most intravascular lymphomas are large B-cell lymphomas, it is an aggressive lymphoma with a poor prognosis. The molecular characteristics are similar to germinal center or post-germinal center B cells. such as diffuse large B-cell lymphoma (DLBCL), which is the most common subtype. Ramkumar et al. found that the Smurf2-YY1-c-Myc axis has become an important regulatory mechanism in suppression of B-cell proliferation and consequently lymphomagenesis (81). The anti-tumor effects of B cells depend mainly on the secretion of anti-tumor-associated antigen (TAA) antibodies and the provision of co-stimulatory signals to TAA-specific CD4+T cells to activate T cells. Kruppel-like factor 4 (KLF4) is a member of the zinc finger-containing KLF transcription factor family involved in regulating apoptosis, proliferation, and differentiation of B-cells and B-cell malignancies (82). Martinez et al. identified two YY1-binding sites in the KLF4 promoter region, and overexpressing YY1 resulted in significant promotion of KLF4 (83). However, whether the regulatory role of YY1 in lymphoma is suitable for the regulation of tumor by B cells in the tumor microenvironment needs to be further confirmed in future studies. the direct regulation of YY1 on tumor immune microenvironment in various tumors are shown in **Table 3**.

**Table 3:** The mechanism of YY1 in the tumor immune microenvironment.

Cancer type	Target gene	Function	Location	Ref.
melanoma	PD1/Lag3/Tim3-up, IL-2/IFN-γ-down	Promotes immune exhaustion	T cell	(62, 63)
glioblastoma	CDK9/up	Suppress interferon response	Cancer cell	(84)
NA	Foxp3/down	Inhibits differentiation and function of Treg cells	Treg cell	(69)
lung cancer	FGL1/up	Inducing T cell apoptosis	T cell	(67)
melanoma	PLZF/up	Promotes iNKT cell development	iNKT cell	(85)
NA	IFN-γ	NA	Jurkat-T cell	(86)
lymphoma	KLF4/up	Promotes tumor progression	B cell	(82, 83)
NA	CXCR4/down	Promote the phagocytosis of macrophages	Macrophage	(87)
NA	COX-2/up	NA	Macrophage	(88)
breast cancer	BRCA1/down	Promotes tumor progression	Cancer cell	(89)
DLBCL	c-Myc/up	Promotes B cell proliferation	B cell	(81)

#### 3.2.3 Other immune-related cells

The TME has appreciable numbers of immune cells, including adaptive immune responses, such as T cells and B cells, and innate immune cells, such as macrophages and neutrophils (90, 91). Tumor-associated macrophages (TAMs) infiltrate tumor tissue and play an essential role in the TME (92). Joo et al. reported that YY1 and STAT1 were upregulated in macrophages stimulated by oxidized LDL and subsequently were translocated into the nucleus to upregulated miR-29a, thereby reducing proinflammatory cytokine secretion and dampening inflammatory responses in macrophages. Cyclooxygenase 2 (COX-2) has been reported to be involved in inflammatory environments and lung cancer pathogenesis, and lipopolysaccharide treatment induced YY1 binding to the homologous site of endogenous COX-2 promoter YY1 and enhances its transcriptional activity in macrophages. Mechanically, YY1 was reported to interact with p300 and HDAC1/2 to affect the acetylation status of YY1. However, lipopolysaccharide treatment disrupted this interaction and binding to the COX-2 promoter. Indicated that it may be a competitive relationship between YY1 and COX-2 promoter, p300 and HDAC1/2 after lipopolysaccharide treatment (88). In addition, Xu et al. indicates that miR-301a/YY1/CXCR4 signaling pathway plays an important role in macrophage migration and phagocytosis (87). Hence, these results strongly suggest that YY1 is critically important for the tumor immune microenvironment.

### 3.3 Role of YY1 in tumor metabolism

As we all know, one of the critical features of the TME is metabolic abnormalities. Due to the rapid growth of tumors, cancer cells have increased anabolic and energy demands (93, 94). Therefore, extracellular nutrients determine the proliferation and growth rate of tumor cells. However, unlike normal cells, cancer cells have more remarkable metabolic plasticity, which forces them to adapt to poor environmental conditions (95, 96). Tumor metabolism abnormalities occur mainly in tumor cells, and cellular metabolites are a vital part of the TME and play an essential role in the formation of the TME. In addition to tumor cells, the interactions between different cells in the TME shape the unique metabolic characteristics of the microenvironment and maintain tumor growth, such as CAF, immune cells, and stromal cells, but the metabolism of YY1 in these components has rarely been reported. As an essential transcription factor, YY1 was reported to be involved in glucose, glutamine and lipid metabolism by regulating the transcriptional activation and expression of some key molecules related to these metabolic processes.

#### 3.3.1 YY1 in glucose metabolism

Since glucose is the primary energy source of organisms used in aerobic and anaerobic respiration (97, 98), which is maintains a balance between catabolic glycolysis/oxidative phosphorylation (OXPHOS) and anabolic gluconeogenesis/glycogen production (99). Abnormal glucose metabolism is integral to tumor metabolic reprogramming in the TME (100). YY1 is closely related to the glycolysis process. The glucose transporter mediated by the glucose transporter family (GLUT) is a pacemaker of aerobic glycolysis and, thus, is essential for tumor cell metabolism. YY1 may indirectly participate in reprogramming glucose metabolism in tumor cells by promoting the stability of HIF-1α under hypoxic conditions to enhance GLUT1 and GLUT3 expression (101). Wang et al. also reported that YY1 activates GLUT3 transcription by directly binding to its promoter while enhancing aerobic glycolysis in colon carcinoma cells (102). In NSCLC cells, circ_0000517/miR-330-5p/YY1 axis promotes the proliferation, glycolysis, and glutamine catabolism by regulating the expression of glycolysis and glutamine catabolism-related genes HK2, LDHA, ASCT2 and GLS1 (103). LDHA is a glycolytic enzyme that converts glucose-derived pyruvate into lactic acid. YY1 directly bound to the promoter LDHA, and overexpression of LDHA reverses the inhibitory effect of sh-YY1 on aerobic glycolysis and proliferation of neuroblastoma cells, indicating that YY1 induces aerobic glycolysis and proliferation (104). Glycolysis-related genes are critical to the glycolysis process. In neuroblastoma (NB), researchers searched public neuroblastoma datasets to identify transcriptional regulators and their glycolysis-related genes. They found that the zinc finger domain of YY1 binds a 21 amino acid peptide encoded by an open reading frame upstream of Myeloid zinc transcription factor 1 (MZF1), called MZF1-upep. MZF1 has been found to exert independent prognostic and promotes the aerobic glycolysis and tumor progression. Notably, the combination of these leads to decreased transcriptional activity of YY1 and inhibited transcription of MZF1 and downstream glycolytic genes HK2 and PGK1, therefore inhibiting aerobic glycolysis and NB progression (105). Hepatitis B virus (HBV) pre-S2 mutant induces HCC by inducing endoplasmic reticulum stress and activating the mTOR signaling pathway. Research has found that pre-S2 mutants initiate the glycolytic *via* activate mTOR signal pathway and activate SLC2A1 in YY1-dependent transcription, contributing to increased aerobic glycolysis, glucose uptake, lactic acid production and release in advanced tumorigenesis of HCC (106). In general, the above studies confirmed that YY1 alters the hypoxic microenvironment of tumor cells by regulating the whole process of glycolysis through different molecular mechanisms in various tumors, thus promoting tumorigenesis.

Since Otto Warburg discovered the characteristic of high-level glycolysis in tumor cell metabolism programming, which not only upregulates the adaptive potential of tumor cells to fluctuating oxygen tension but also produces glycolytic intermediates and increases the pentose phosphate pathway (PPP) (107–109), the primary significance of the PPP can generate nicotinamide adenine dinucleotide phosphate (NADPH) and a variety of monosaccharides. NADPH is the only enzyme specifically used to produce reactive oxygen species (ROS) and is essential for cellular antioxidant defense (110, 111). A recent study by Wu et al. identified that YY1 directly binds to the promoter of G6PD and promotes its transcription activity and expression, therefore, stimulating the PPP and protecting tumor cell from oxidative stress and inducing tumor progression (112). In conclusion, research on the molecular mechanisms of the G6PD-mediated PPP is closely related to YY1-induced tumor cell proliferation and tumorigenesis.

**Figure 3:**
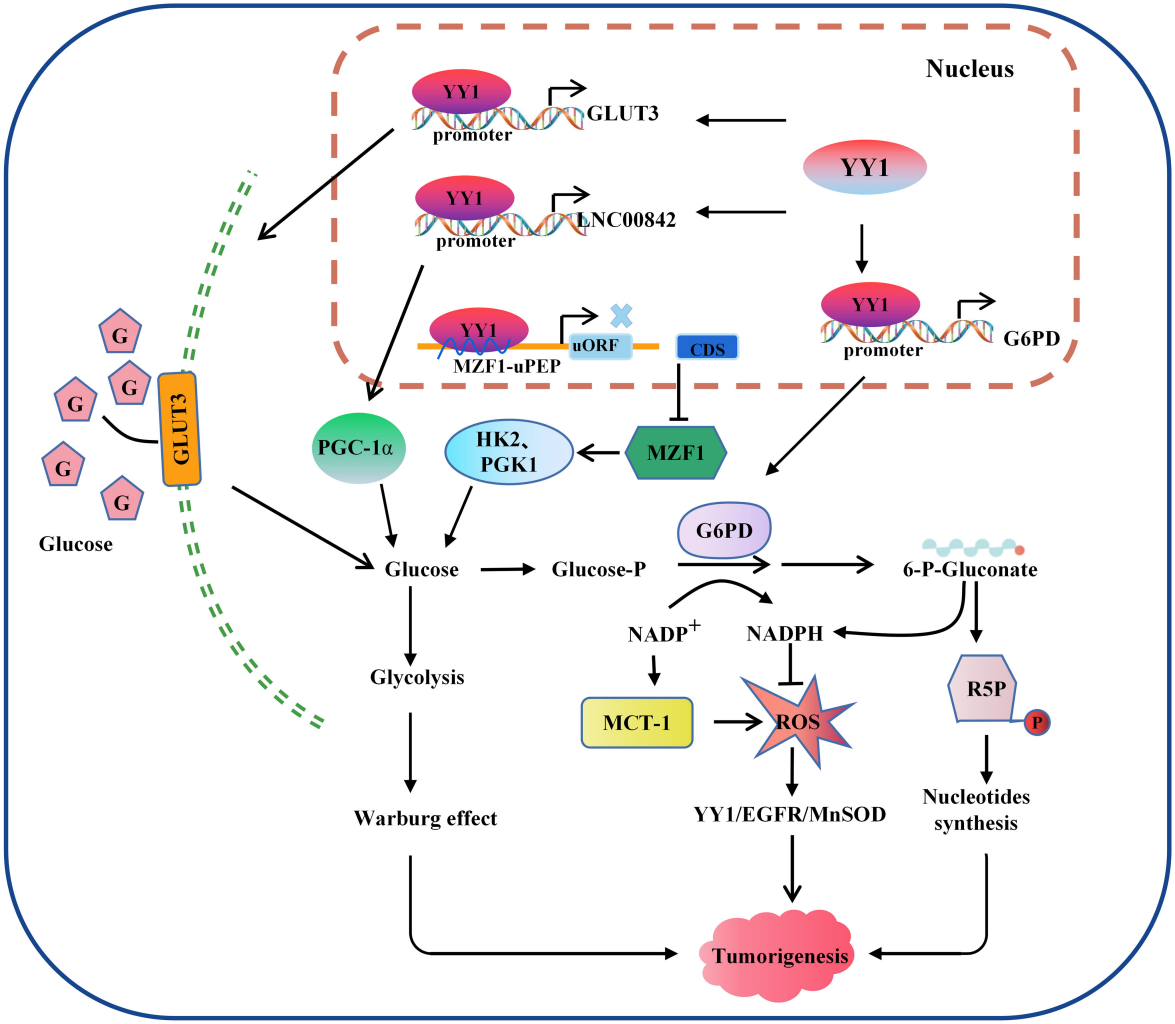
Model of the mechanism through which YY1 regulates glucose metabolism. YY1 is involved in regulating glucose metabolism in tumor cells by affecting the transcription activation of some key molecules. For example, activation of GLUT3 and LNC00842 transcription enhances aerobic glycolysis in tumor cells, while downregulation of YY1 by MZF1 inhibits the transcription of its downstream glycolytic genes HK2 and PGK1, thereby inhibiting aerobic glycolysis. In addition, YY1 activates G6PD transcription and stimulates the pentose phosphate pathway to enhance nucleotide generation and DNA synthesis and reduce intracellular reactive oxygen species levels, thus promoting tumorigenesis. YY1, Yin Yang 1; G6PD, glucose-6-phosphate dehydrogenase; GLUT, glucose transporter; ROS, reactive oxygen species; HK2, hexokinase 2; PGK1, phosphoglycerate kinase 1; R5P, Ribulose 5-phosphate.

One of the typical features of metabolic changes in cancer cells is high levels of oxidative stress, and higher cellular oxidative stress levels favor the activation of glycolysis and promote tumorigenesis by affecting the signal transduction system, depriving them of normal contact inhibition, and promoting invasion and metastasis (113). The functions of these are complementary to each other. Tseng et al. found that p53 accumulation was reduced by MCT-1 overexpression. However, the levels of manganese-dependent superoxide dismutase (MnSOD) were upregulated through the YY1-EGFR signaling pathway, protecting cells from oxidative damage. On the other hand, limiting ROS production and inhibiting YY1 in lung cancer cells prevented MCT-1-induced cell invasiveness and the EGFR-MnSOD signaling pathway (114). However, ROS and YY1 do not have a definite positive association. According to research, low levels of ROS promoted the production of YY1, while high levels of ROS clearly inhibited it. YY1 was recruited to the antioxidant-responsive element binding site when its expression was stimulated, working in concert with other coregulators and then boosted NRF2-mediated ARE transcription, thereby shielding cells from damage by amplifying the antioxidant response (115). Overall, the available data indicate that YY1 can significantly activate cell glycolysis and ROS metabolism and play a critical role in remodeling the TME (**Figure 3**).

#### 3.3.2 YY1 in lipid metabolism

**Figure 4:**
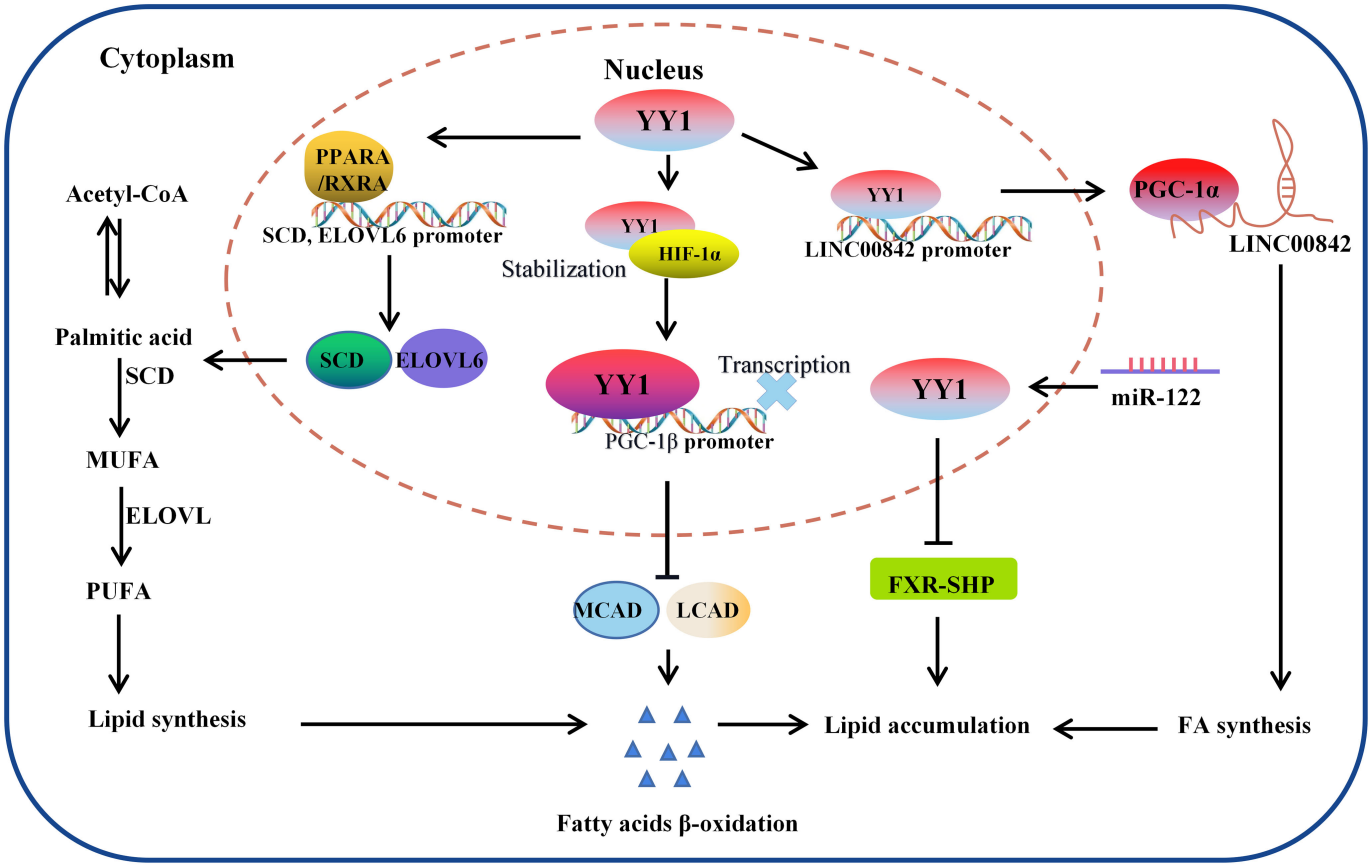
The molecular mechanism of YY1 regulating lipid metabolism. YY1 promotes triglyceride accumulation by regulating lipid metabolism-related molecule transcription. In this process, YY1 suppressed fatty acid β-oxidation, further inhibiting the expression of medium-chain acyl-CoA dehydrogenase (MCAD) and long-chain acyl-CoA dehydrogenase (LCAD) which is the key enzymes necessary for FAO. Additionally, miR-122 could promote lipid droplet formation and triacylglycerol (TG) accumulation *in vitro* by reducing the stability of YY1 mRNA and upregulating FXR-SHP signaling. Furthermore, YY1 also leads to lipid accumulation through the PPARA, RXRA/SCD, ELOVL6 signaling pathway and regulated the expression of LINC00842. LCAD, long-chain acyl-CoA dehydrogenase; MCAD, medium-chain acyl-CoA dehydrogenase; PGC-1β: peroxisome proliferator-activated receptor gamma coactivator-1β; SCD: PGC-1α: peroxisome proliferator-activated receptor gamma coactivator-1α.

It has been widely observed that lipid metabolic reprogramming during cancer development confers cancer cells the ability to survive through enhanced lipid synthesis, storage, and catabolism, even under nutrient-limiting conditions. Abnormal lipid metabolism has always been observed in various tumors, and chronic liver disease and nonalcoholic fatty liver disease (NAFLD) are closely related to lipid homeostasis disorder. Accumulating evidence suggests that the hallmark feature of NAFLD is an increase in intrahepatic TG content (116). To investigate the crosstalk between lipid metabolism and YY1 in liver cancer cells, the authors found that several key transcription factors of lipid metabolism and their coactivators were significantly reduced in YY1-knockout HCC cells, such as PPARA/RXRA complex member CHREBP, SREBF2 and FXR and HNF family member HNF4A, FOXA1 and FOXA2 (117–119). Moreover, silencing YY1 suppresses the expression of crucial transcription factors markedly reduced their cooperation at various regulatory levels and various regulatory regions, which are involved in hepatic lipid metabolism regulated by these proteins. Especially the expression of SCD and ELOVL6, which encode key enzymes for adipogenesis, is regulated cooperatively by YY1 and PPARA/RXRA complexes on their promotors (120). In normal cells, cellular fatty acid concentration balance is controlled by the normal synthesis and breakdown of fatty acids. But tumor cells modify lipid metabolism by speeding up fatty acid synthesis while blocking fatty acid breakdown, leading to intracellular lipid buildup, in order to meet the demands of their rapid multiplication and growth. Li et al. demonstrated that YY1 was crucial for alterations in lipid metabolism in HCC cells because it suppresses the expression of the PGC-1β, which is a transcriptional activator of MCAD and LCAD that boosts both enzymes’ expression. The MCAD and LCAD are key enzymes required for FAO and crucial for fostering carcinogenesis (121). Therefore, YY1 upregulation prevented fatty acid β-oxidation, which increased triglyceride levels and lipid buildup in HCC cells and demonstrated the promising potential to accelerate the growth of tumors. Wu et al. also demonstrated that miR-122 could promote lipid droplet formation and triacylglycerol (TG) accumulation *in vitro* by reducing the stability of YY1 mRNA and upregulating FXR-SHP signaling. Suggesting that YY1 plays an essential role in regulating the accumulation of hepatic lipids, especially hepatic triglycerides (TGs) in the TME (122) (**Figure 4**).

#### 3.3.3 YY1 in glutamine metabolism

Glutamine is an essential amino acid to maintain normal cell biological function, including biosynthesis, cell signaling, and preventing antioxidant damage (100). Of note, glutamine metabolism can provide the energy and biomacromolecules required by tumor cells for rapid growth and proliferation, helping them maintain intracellular redox homeostasis and intracellular signaling (123), and their anabolic/catabolic actions are dysregulated in cancer. Following glucose and fatty acid metabolism, oncogene-mediated abnormal glutamine metabolism in tumor cells has emerged as a new energy source (124). The researchers analyzed the effect of the YY1 on biological processes using the DAVID method, and these results suggested that the promotion of glutamine metabolism by YY1 may promote the progression of ESCC by affecting cancer pathways. Furthermore, the expression of key rate-limiting enzymes ASCT2, GLS and GLUT1 showed an upward trend and led to glutamine uptake and production were significantly increased. Briefly, these data support a model that YY1 promotes glutamine metabolism and regulates the development of ESCC (125). We also noted that YY1 promotes the expression of vital rate-limiting enzymes HK2, LHDA, PCNA, ASCT2 and GLS in A549 cells. Accordingly, YY1 obviously promoted glucose-to-glutamate conversion (103). In summary, these studies illustrated the role of YY1 in glutamine metabolism in the tumor, and the role of YY1 and its molecular mechanism on tumor metabolism is shown in **Table 4**.

**Table 4:** The function and mechanism of YY1 on tumor metabolism.

Cancer type	Target gene/regutation	Signal pathway	Metabolism type	Ref.
CRC	CLUT3/up	YY1/CLUT3	aerobic glycolysis	(102)
Neuroblastoma	MZF1/down	YY1/MZF1/HK2- PGK1	aerobic glycolysis	(105)
HCC	MYC/up	MTOR/EIF4EBP1/YY1/MYC/SLC2A1	aerobic glycolysis	(106)
NSCLC	NA	circ_0000517/miR-330-5p/YY1	glycolysis glutamine	(103)
CRC	NA	miR-31HG/miR-361-3p/YY1	glycolysis	(54)
neuroblastoma	LDHA/up	YY1/LDHA	aerobic glycolysis	(104)
multiple tumors	G6PD/up	YY1/G6PD	pentose phosphate pathway	(112)
lung cancer	MCT-1/up	YY1/MCT-1/EGFR/MnSOD	oxidative metabolism	(114)
HCC	PPARA/up	YY1/PPARA/SCD, ELOVL6	lipid metabolism	(120)
HCC	PGC-1β/down	YY1/PGC-1β/MCAD, LCAD	lipid metabolism	(121)
pancreatic cancer	LINC00842/up	YY1/LINC00842/SIRT1/PGC-1α	lipid metabolism	(126)
esophageal carcinoma	ASCT2, GLS, GLUD/up	YY1/ASCT2, GLS, GLUD1	glutamine	(125)
melanoma	TGF-β1/down	YY1/TGF-β1/Smad 2	glutamine	(127)

## 4 Clinical potentials of YY1 and the microenvironment

### 4.1 The clinical value of YY1 in cancer diagnosis

In recent years, molecular diagnosis has become an important strategy for clinical tumor diagnosis and prognosis evaluation based on the expression specificity and clinical relevance of YY1 in different tumors. On the one hand, YY1 is highly expressed in prostate cancer, gastric cancer, ovarian cancer, breast cancer, multiple myeloma, liver cancer, and lung cancer etc. It especially shows increased expression in metastases compared with primary tumors, suggesting that high expression of YY1 may be a molecular marker for early diagnosis and prognosis evaluation of these tumors. On the other hand, the expression of YY1 was decreased in PDAC, NPC, pediatric osteosarcoma, etc., and negatively correlated with its clinical progression and a poor prognosis, suggesting that the low YY1 expression predict poor prognosis of these tumors. Tumor cells evade the immune response by producing a TME that suppresses the immune response, specifically a TME involving the T cell checkpoint receptor PD-1. It is a mechanism that prevents overstimulation of the immune system. Studies have shown that YY1 expression is positively correlated with PD-L1 expression, so elevated YY1 expression is expected to be a molecular marker for diagnosing T cell-mediated tumor immune escape (66). In addition, the expression level of YY1 in Burkitt lymphoma and DLBCL is higher than that in normal B cells and low-grade lymphoma (128). As mentioned above, YY1 may be a valuable diagnostic and prognostic marker for chemotherapy or immunotherapy regimens.

### 4.2 YY1 is an important molecular target for tumor therapy

The TME is a facilitator of tumor drug resistance (129), and the TME influences the progression and metastasis of solid tumors. Most current therapies target the tumor cells themselves, ignoring the local microenvironment around them. However, it is becoming clear that the most effective approach for cancer treatment involves therapies targeting both tumor cells and the TME (130, 131). Based on the functions of YY1 in tumor angiogenesis, metabolism and tumor-related microenvironment reconstruction, targeting YY1 are expected to become an important molecular strategy for tumor therapy (132). Previous studies have proven that many clinical drugs targeting YY1 have sound therapeutic effects on YY1-mediated tumor drug resistance or malignant progression. For example, the clinical drug oxaliplatin was reported to suppress colon carcinoma cell proliferation and angiogenesis by inhibiting the YY1/GLUT3 axis (102). Another study proved that YY1 plays an important role in angiogenesis and bevacizumab resistance by inducing VEGFA transcriptional activity and expression, and YY1 may be a potential molecular therapeutic target for anti-angiogenic therapy in HCC (46). In addition, cisplatin has always been one of the drugs of choice for head and neck squamous cell carcinoma (HNSCC), but most tumors that are initially responsive to cisplatin later acquire resistance. Zhao et al. found that targeting YY1 or PP2A enhanced the efficiency of cisplatin chemotherapy in HNSCC (133). Therefore, YY1 plays a crucial role in regulating TME-mediated chemoresistance in tumor cells, which has important implications for diagnosing and treating patients.

Immunotherapy has become a mainstay of cancer treatment in many malignancies, and clinical evaluation confirmed that the expression of the oncogene YY1 was upregulated in PD-1^+^ T cells infiltrating lymphocytes in tumors, suggesting that YY1 may hinder T-cell-mediated tumor immunotherapy, especially since the effect on PD-1/PD-L1 is not fully blocked. Therefore, exploring the underlying mechanism of regulating PD-L1 expression on tumor cells is a way to make tumor cells effective for PD-1/PD-L1 antibody therapy. Multiple signaling crosstalk pathways have been reported to be involved in the regulatory relationship between YY1 and PD-L1 (66). In addition, remodeling the immunosuppressive microenvironment and enhancing immunotherapy response are effective strategies for the clinical treatment of cancer. Qiu et al. confirmed that YY1-dependent glioblastoma stem cells were sensitive to the CDK9 inhibitors (Alvocidib and Dinaciclib), and the combination of Alvocidib and anti-PD-1 can more significantly reshape the tumor immune microenvironment, enhance the immunotherapy response and inhibit the occurrence of glioma (84). Based on these findings, combining strategies targeting YY1 and the above pathways may enhance cell-mediated antitumor cell responses and reverse the resistance observed with checkpoint inhibitors alone. Therefore, YY1 is a critical target for immunotherapy and chemotherapy of cancers. However, YY1 acts as a tumor suppressor gene in pancreatic cancer and nasopharyngeal cancer, and specific clinical drugs targeting YY1 have not yet been discovered. We look forward to having made better progress in improving tumor treatment efficacy in the future.

## 5 Conclusions and perspectives

YY1 is a critical transcription regulator and is closely related to the remodeling and regulation of the TME, and it has two distinct expression patterns (high or low) in different tumor types. It is upregulated in most types of tumors, such as prostate cancer, gastrointestinal cancer, ovarian cancer and breast cancer, and downregulated in a few tumors, including nasopharyngeal carcinoma, thus, it plays a dual role as an oncogene or tumor suppressor in tumorigenesis and tumor progression. YY1 plays critical roles in tumor angiogenesis, glucose metabolism, lipid metabolism, and immune regulation, which provides an important perspective for understanding the mechanism YY1 is involved in tumorigenesis and development. Based on the specific expression patterns of YY1 in tumors and its role in the formation and regulation of the TME, targeting YY1 and its related TME would be a potential molecular strategy for the clinical diagnosis and treatment of tumors. Although YY1 as a target has demonstrated a valuable application prospect in the diagnosis and treatment of tumors, and certain drugs or inhibitors could indeed alleviate the clinical malignant phenotype or drug resistance of tumor patients by reversing YY1 expression, there is still no specific and inhibitor or targeted drug to YY1 used for the clinical treatment of tumors, further efforts are needed for researchers to develop specific inhibitors or drugs targeting YY1.

## Author contributions

MZ, ML, WX, FT and GL designed and conducted this study. ML and JW drafted the manuscript. CX, XZ, SC, LZ, YD and HD revised the manuscript. All authors contributed to the article and approved the submitted version.
